# Local Anæsthesia and Atomization of Liquids

**Published:** 1869-02

**Authors:** 


LOCAL ANÆSTHESIA AND ATOMIZATION OF LIQUIDS.

We are under many obligations to Messrs. GODMAN & SHUTLEFF, Nos. 13 and 15 Tremont street, Boston, for a specimen of their Apparatus for the Inhalation of Atomized Liquids; for their instrument for treating diseases of the Nasal Cavity.—These instruments are admirably gotten up, and most convenient and useful in the treatment of diseases for which they are intended. We regret that we have misplaced the cuts of the several instruments, as we would take pleasure in presenting them to the profession, that they might more fully understand their several uses and the advantages to be derived from them in practice. These gentlemen also have on hand a large supply of Rhigoline for Local Anæsthesia, Stethoscopes, Laryngoscopes, Endoscopes, Skeletons, Manikins, Charts, etc., etc.; besides a full supply of Surgical and Dental Instruments, and are ready to supply the profession at reasonable prices.

